# Supplementary figures and images for: Depth in convolutional neural networks solves scene segmentation
Source: PLoS Comput Biol. 2020 Jul 24;16(7):e1008022. doi: 10.1371/journal.pcbi.1008022 (PMC7406083; doi:10.1371/journal.pcbi.1008022)

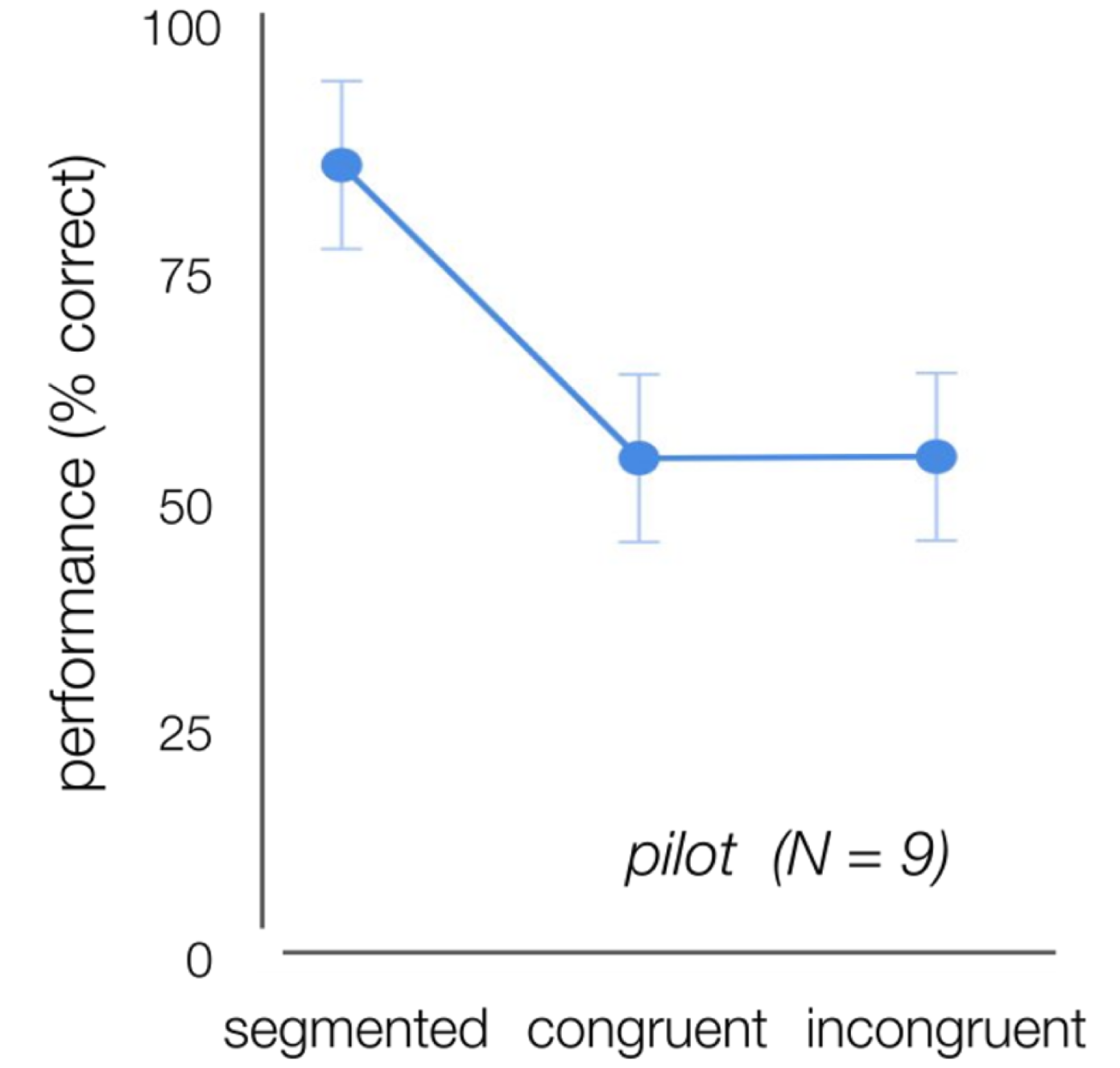

Supplement: S1 Fig — Performance was higher for the segmented condition compared to congruent and incongruent. (TIF) [file pcbi.1008022.s001.tif]
