# Supplementary material for: Depth in convolutional neural networks solves scene segmentation
Source: PLoS Comput Biol. 2020 Jul 24;16(7):e1008022. doi: 10.1371/journal.pcbi.1008022 (PMC7406083; doi:10.1371/journal.pcbi.1008022)
Supplement: Attachment — Submitted filename: ResponseToReviewers_ImplicitSceneSegmentation.pdf [file pcbi.1008022.s002.pdf]

Editor's comments:

Thank you very much for submitting your manuscript "Depth in convolutional neural networks solves scene segmentation" for consideration at PLOS Computational Biology.

As with all papers reviewed by the journal, your manuscript was reviewed by members of the editorial board and by several independent reviewers. In light of the reviews (below this email), we would like to invite the resubmission of a significantly-revised version that takes into account the reviewers' comments.

We cannot make any decision about publication until we have seen the revised manuscript and your response to the reviewers' comments. Your revised manuscript is also likely to be sent to reviewers for further evaluation.

We thank the Editor for giving us the opportunity to improve our paper. We have thoroughly revised the manuscript to address the concerns of both reviewers. We truly believe the manuscript has substantially improved by incorporating the reviewer’s suggestions, and we hope you will now consider it suitable for publication. We have made the following major changes:

- As proposed by Reviewer 1, we included different CORnet architectures (feedforward and recurrent) to support our claims about recurrent processing.
- To make the comparison between humans and DNNs more valid, and to estimate the reliability of the effects in experiment 1, we showed 38 different subsets of the stimuli to the DNNs, each subset consisting of the same number of images per category and condition that the human observers were exposed to.
- Related to the previous point, both reviewers asked for statistical analyses of DCNN performance: We have incorporated the statistical analyses for the DCNNs and have revised the manuscript accordingly. We have integrated parts of the Methods within the Results section to make the results more understandable.
- We have updated existing Discussion paragraphs to provide more context for our interpretations on how, and when object information is differentiated from the backgrounds they appear on, as suggested by Reviewer 2.
- As proposed by Reviewer 2, we included a visualisation of the filter activations of each convolution layer.

All changes are detailed below in a point-by-point response, with the reviewer comments appearing in black italic font and our responses in regular blue font.

Reviewer's Responses to Questions

Comments to the Authors:
Please note here if the review is uploaded as an attachment.

Reviewer #1:

Seijdel and colleagues conducted a study to test whether object information is differentiated from backgrounds and asked how it may work. The authors performed a behavioral experiment on humans and experiments on DNNs. The main finding is that DNN depth facilitates the segmentation of an object from a background. The main advancement in the study is showing how humans and DNNs differ and how deeper DNN may perform better on this task.

I see several issues with this manuscript that I would invite the authors to address.

We thank the reviewer for their comments and feedback on our manuscript and address the reviewer's concerns below.

1. The authors try to make claims about the recurrence being important for segmentation as it has been shown that deep DNNs can somewhat approximate recurrent DNNs. However, a more direct way to make this argument would be to test a recurrent DNN. One class of recurrent models (CORnet-R and CORnet-S) can be easily tested by extracting the activations using this Github repo: https://github.com/dicarlolab/CORnet. Additionally, other labs like Thomas Serre, Dan Yamins, Tim Kietzmann and Niko Kriegeskorte have recurrent models that could also be tested. If this manuscript is making claims about recurrent processing at least one recurrent model should be tested.

Response: We agree with the reviewer that in order to make claims about recurrent processing, testing recurrent models is important. In our approach, we build heavily on previous literature that shows that very deep ("ultra-deep") residual networks are mathematically equivalent to a recurrent neural network unfolding over time, when the weights between their hidden layers are clamped (Liao & Poggio, 2016) and suggests that deeper DCNNs might be approximating "unrolled" versions of recurrent circuits of the ventral stream (Kar, Kubilius, Schmidt, Issa & DiCarlo, 2019). We chose ResNet architectures because they can be scaled up and down in size (depth) easily, by adding or removing their basic building blocks. This approach allowed us to investigate the effect of network depth (adding layers) while keeping other model properties as similar as possible.

However, in order to further support our claims about recurrent processing, we have followed the reviewer's suggestion and tested three different architectures from the CORnet model family; CORnet-Z (feedforward), CORnet-RT (simple recurrent within areas) and CORnet-S (recurrent with skip connections) (Kubilius, Schrimpf, Nayebi, Bear, Yamins & DiCarlo, 2018; Kubilius, Schrimpf, Kar, Rajalingham, Hong, Majaj, … & Nayebi, 2019).
From these three networks, CORnet-S is the highest-performing network. Inspired by ResNets, two more convolutions are stacked on top of CORnet-RT's circuit (each followed by a normalization and nonlinearity), and a skip connection is included.

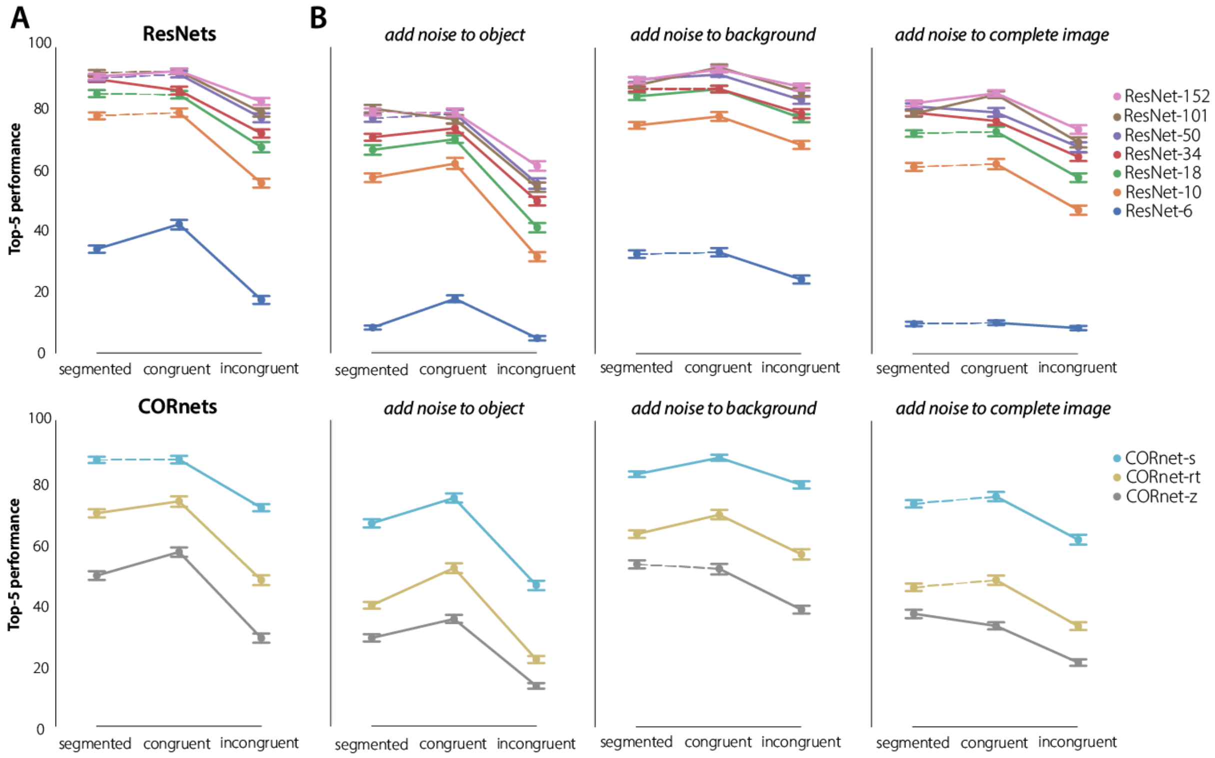

For all CORnets, performance was lowest for the incongruent condition. This effect was particularly strong for the smallest (feedforward) architecture, CORnet-Z. For this architecture, performance was also better for the congruent condition compared to the segmented condition, suggesting that there is 'leakage' of the natural (congruent) background in the features for classification. Results from CORnet-RT indicated a similar pattern, but with an overall increase in classification performance. For CORnet-S, however, the presence of a background (segmented, congruent or incongruent) had a less strong influence on behavior, similar to the 'ultra-deep' ResNets.

The shift in performance from CORnet-Z to CORnet-S shows the same pattern as the shift from ResNet-6 to ResNet-18. This overlap suggests that the pattern we observe in ResNets can indeed be approximated by recurrent networks. Because the different CORnet models did not only differ with respect to 'recurrence', but also contained other architectural differences (CORnet-Z not only is feedforward, but it is also shallower than CORnet-S), the differences between the networks could stem from the difference in information flow (feedforward vs. recurrent), or from the different amount of parameters in each network.
Taking the results from the ResNets and CORnets together, these findings suggest that one of the ways in which network depth improves object classification, is by learning how to select the features that belong to the object, and thereby implicitly segregating the object features from the other parts of the scene.

Action: Following suggestions by Reviewer 1, we have incorporated results from feedforward and recurrent CORnet architectures in our manuscript. For consistency, because CORnet is defined in PyTorch, we have also re-analyzed the ResNets using Pytorch, in order to evaluate all networks (ResNets and CORnets) in the same framework. We have updated the Results and Discussion sections accordingly.

2. Differences between DNNs trained with segmented vs unsegmented images seem to be very small. As there are differences between DNNs trained multiple times I would like to ask the authors to train each DNNs tested at least three times with different initialization conditions to see whether the effects of visual training diet will be indeed significant across multiple initialization conditions of the models. All DNN figures should be based on multiple initialization conditions of the models, rather than just one, with error bars expressing standard deviation across initialization conditions.

Response: The results the reviewer is referring to (from experiment 2) were based on multiple (ten) initializations with different seeds. Although it was difficult to see, the original plots contained error bars expressing the standard error of the mean. For clarification, following the reviewer's suggestion, we now include error bars expressing the standard deviation across these ten initialization conditions.

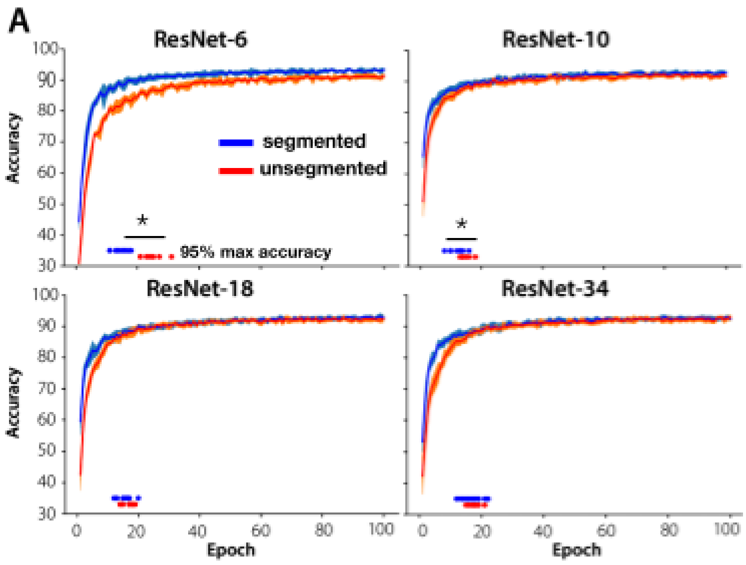

Mann-Whitney U tests comparing the 'speed of convergence' indicate that the effects of visual training diet (segmented vs. unsegmented) are significant across multiple initialization conditions of the networks, especially for the more shallow networks (U=0, $p < .001$; U=20.0, $p = 0.119$ for ResNet6 and ResNet10 respectively). For this analysis, the speed of convergence was defined as the first epoch at which 95% of the maximum accuracy was reached.

Action: We have updated the Methods and Results sections to clarify the effect of visual training diet (segmented vs. unsegmented) across multiple initialization conditions of the models.

3. Is seems that the numbers of images shown to participants and DNNs were different:
For humans: "243 images were generated for the actual experiment"
For DNNs: "810 images with a congruent background, 810 with an incongruent background and 270 images with segmented objects"
Why DNNs did not see the same images as humans to make the comparison between humans and DNNs more valid?

Response: For human participants, each exemplar was presented only once (27 object categories * 9 exemplars = 243 trials) to exclude any repetition- or learning effects. We reasoned that this was not necessary for the DCNNs, as they don't 'remember' what they see during testing. Therefore, initially, all images were used to assess object recognition performance for the DCNNs.

However, we agree with the reviewer that, to make the comparison between humans and DNNs more valid, it is important to keep the testing conditions as similar as possible. This point also addresses point 5 of the reviewer, in which the reviewer asks for statistical results for the DCNNs. Multiple runs on different image selections allow us to estimate the reliability of the effects in our experiment by indicating a range of DCNN accuracies (error bars) that we can use for statistical analysis. Therefore, to make the comparison between humans and DNNs more valid, and to estimate the reliability of the effects in experiment 1, we now show 38 different subsets of 243 stimuli to the DNNs, each subset consisting of the same number of images per category and condition that human observers were exposed to (81 per condition, 3 per category).

Action: We have updated our Methods and Results section.

4. Figure 1D – What are the error bars? Std across participants? It should be stated in the figure legend.

Response: As part of the revisions made to accommodate new analyses suggested by Reviewer 2, this part of the Results section has been revised. In the updated Figure 1, error bars represent 95% confidence intervals. We apologize for the missing information and have updated the figure legend.

5. Figure 2B – I would like to know which differences between the segmented, congruent and incongruent conditions are significant (like in Figure 1B for human participants).

Response: We agree with the reviewer and have followed their suggestion. As described under point 3, multiple runs on different image selections allowed us to perform statistical analyses on DCNN performance.

6. Figure 5B - What do error bars represent? It should be stated in the figure legend.

Response: We apologize for the missing information. In experiment 2, we reinitialized the networks ten times with different seeds to obtain statistical results. The error bars in Figure 5B represent the standard error from the mean. In the revised plots, we have visualized the individual data points for each of the initializations to increase interpretability.

7. In the discussion section there is a part about attention that I find quite confusing: “It also suggests that, with adequate deployment of attention, a deeper network is not necessary to recognize the object “. The authors should more clearly define what they mean by attention, how attention differs from recurrent processing, and how relevant it is to mention it here.

Response: We understand the confusion. For humans, we would argue that this is indeed not relevant. In the visual routines framework, increased perceptual grouping or ‘stitching together object features’ via slow recurrent processes is often thought to be the same thing as object-based attention (see e.g., Jeurissen et al., 2016).

For the networks, however, the crucial aspect is that the network should be able to select features that belong to the object, while at the same time being able to ignore or suppress features from other parts of the scene. With the statement about attention, we meant to clarify that network depth (or recurrence) is not the only way or mechanisms this goal can be achieved within the network. DCNNs armed with attention mechanisms, for example, might be able to solve the same ‘problem’ by attending to what's relevant (the object), while disregarding other parts of the scene. However, we agree with the reviewer that this statement is not directly relevant to mention in the Discussion section, therefore we have decided to remove this statement.

Action: We have removed this statement.

Reviewer #2:

The authors have explored a timely topic re the depth of the DCNNs and the effect of depth on automatic scene segmentation. It was further interesting to see how this is potentially linked to the hierarchy of vision and the two modes of processing in the brain: feedforward vs. recurrent. I like their careful consideration of congruent and incongruent background, while some of the key previous literature has unfortunately ignored this important parameter by placing objects on incongruent backgrounds, when defining the core object recognition (see for example ’How does the brain solve visual object recognition?’ Neuron, 2012).

We thank the reviewer for their positive comments on our manuscript. We are glad to see that they appreciate the consideration of congruent and incongruent backgrounds, and address the reviewer’s concerns below.

Major comments:

-Not sure what is the journal requirements, but starting off with the actual results without explaining the experiment itself was confusing. So please either move the methods before the results section; or otherwise integrate some of the method within the results (e.g. explain what experiment 1 is before jumping into the accuracy of participants in a task that is not explained before), and keep further details for the method.

Response: We agree that the opening of the Results section did not provide sufficient context for the reader independent of the Methods about the task. In our revised manuscript, we integrated parts of the Methods within the Results, as the reviewer suggested.

-Figure 1, panel D: Please use non-parametric tests for comparing human accuracies (e.g. bootstrap of participants) —I am not convinced by the ANoVA. Do report the stats for all the

pairwise comparisons. And explain what the error bars are ? Standard error? Std? Confidence interval ..? (ideally you would want to report 95% confidence intervals)

Response: Following the suggestion of the reviewer, we have updated our results for comparing human accuracies. A non-parametric Friedman test differentiated accuracy across the three conditions (segmented, congruent, incongruent), Friedman's Q(2) = 74.053, $p < .001$. Post hoc analyses with Wilcoxon signed-rank tests indicated that participants made fewer errors for segmented objects, than the congruent, W = 741, $p < .001$, and incongruent condition, W = 741, $p < .001$. Additionally, participants made fewer errors for congruent than incongruent, W = 729, p < .001. We have updated the Results section accordingly. In the updated Figure 1, error bars indicate 95% confidence intervals.

-Figure 2, panel B is one of the key results/figures, based on which most of arguments in the manuscript are formulated. However the results (and claims) here are missing a proper statistical support. E.g. it is said that 'For shallow networks, performance is better for the congruent than for the incongruent condition'. What are the statistical analysis that support this argument? I suggest a non-parametric statistical test here to see if indeed the performance of shallower resnets is higher in congruent compared to incongruent —and report the p-value. Also consider multiple comparison correction (e.g. FDR). And similarly all other claims through out the paper that are related to the results of this figure need to be backed statistically.

Response: As part of the revisions made to accommodate new analyses suggested by Reviewer 1, we showed 38 different subsets of 243 stimuli to the DNNs, each subset consisting of the same number of images per category and condition that human observers were exposed to (81 per condition, 3 per category). Multiple runs on different image selections allowed us to estimate the reliability of the effects in our experiment by indicating a range of DCNN accuracies (error bars) that we could use for statistical analysis. Following the procedure for comparing human performance, a non-parametric Friedman test differentiated accuracy across the three conditions (segmented, congruent, incongruent) for all networks. Using Post Hoc Wilcoxon signed-rank tests with Benjamini/Hochberg FDR correction, differences between the conditions were evaluated for all networks (significant differences are indicated with a solid line).

Action: We have incorporated the statistical analyses for the DCNNs in the Methods and Results sections and have revised the manuscript accordingly.

-Figure 3, I could not find a good explanation of how interference (y-axis) is defined here. Please make sure this is explained in the figure legend and the method section.

Response: We apologize for the lack of detail in this reported analysis. For this analysis, images were occluded by a gray patch, sliding across the image in 32 pixel steps. Interference was defined as the relative change in activation (compared to the original image), after occluding pixels in the specific location of the patch. Finally, two values, one for the object and one for the background, were obtained by averaging the 'interference' (change in the feature map) across pixels belonging to either the object or the background.

We chose the term ‘interference’ because it indicates to what degree occluding a certain region i*nterferes* with classification. However, the comment of the reviewer made us realize that ‘interference’ might be a confusing term, and ‘*importance’* might be more intuitive. If occluding a certain region interferes with classification to a higher degree, this region is considered *important* for classification. Therefore, in the revised manuscript we refer to the *‘importance’* of a certain region of the image.

Action: We have edited the legend of Figure 3 and the Methods section to clarify our definition of ‘*importance’* (previously ‘interference’)

-Page 12: “Models trained on segmented objects achieve better classification accuracy in the early stages” . There is no statical support for this statement (and the difference —by eyeballing— seems to be negligible)

Response: With this statement, we meant to emphasize that networks trained on segmented objects reach convergence earlier than networks trained on unsegmented objects. However, we agree that this is not directly tested. Therefore, we have performed an additional analysis to evaluate differences in classification accuracy in early stages. Mann-Whitney U-tests comparing the average accuracy of the first 10 epochs for networks trained on segmented vs. unsegmented objects indicated significant differences across multiple initializations of all networks (Mann-Whitney U-statistic: $U=0.0$, $p<.001$ for ResNet 6, 10, 18 and 34).

- In the discussion, would be good to further explain and give insights that based on the results of this study, how many layers is deep enough for segmentation and give a high-level summary of what you promised in the abstract: “how, and when object information is differentiated from the backgrounds they appear on"

Response: The reviewer brings up two relevant and important questions: 1) based on our findings, how many layers is deep enough for segmentation, and 2) if we can provide a high-level summary of the insights based on the results, regarding the depth and mechanisms underlying figure-ground segmentation.

The current results suggest that there is no discrete ‘moment’ at which segmentation is successful or ‘done’. What the results do show, however, is that, with an increase in network depth, there is a better selection of features that belong to the target object (vs. the background), resulting in higher performance during recognition. Thus, more layers are associated with ‘more’ or better segmentation, by virtue of increasing selectivity for relevant constellations of features. The current results also suggest that this occurs *implicitly* as a function of network depth, without the need for an explicit process in which certain elements of an image are grouped by a labelling process.

Action: we have added a new paragraph to the Discussion section to provide more context for our interpretations.

-It can very well enrich the paper if you provide visualisation of the deep net layers; and give an idea re the extracted features in each of the scenarios you lay out in experiment 2.

Response: We thank the reviewer for this suggestion, which we now include in the revised manuscript. To visualise the filter activations of each convolutional layer, we extracted all the filter activations from the different layers (one 2D-array per filter) for a specific image. Then, for each layer, we summed the absolute values of those arrays together. Visualizing the filter activations of each convolution layer of the networks provides us with heatmaps that show features of a given image, which a corresponding filter attends to. This gives us an idea of which parts of the image contained the most important features for classification.

Looking at the heatmaps of networks trained on segmented vs. unsegmented data, we see that the heatmaps of the networks trained on segmented objects contain no background activations. For networks trained on unsegmented objects (full images), however, we see that the backgrounds are gradually suppressed inside the network. This indicates that the networks learn to attend to important features and by extent to the objects in the images and almost eliminate completely the influence of the background, when the depth (capacity) of the network is sufficient. This suggests that the network learns to segment the objects before classifying. Note that the lightest parts of the heatmaps shown in the figures are the most important features for the classification.

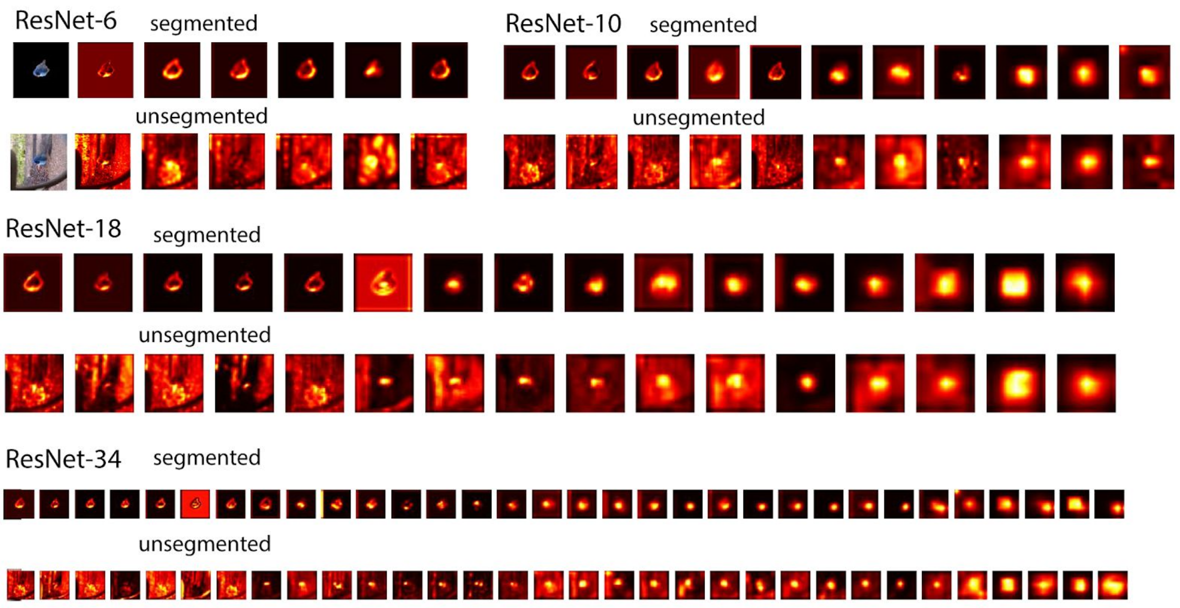

Action: we have included a visualisation of the filter activations of each convolution layer. We have updated the Results section accordingly.

Minor:

-The link to the code and data (on the cover page) is broken. I could find the right page by google, but please update the hyperlink.

Response: We apologize for the oversight and have updated the hyperlink.

-Page 3, “Disruption of visual processing beyond feed-forward stages (e.g. >220 ms after stimulus onset, or after activation of higher order areas)”. : Most of the feedforward processing is done primarily within the first 150 ms after the stimulus onset. 220 ms is not accurate . Please see Liu et al. Neuron (2009), Cichy et al. , nature-neuro (2014), or Khaligh-Razavi et al. JoCN (2018)

Response: In this statement, we refer to a study in which the authors manipulated visual activity ~220 ms using TMS (Camprodon et al., 2013). However, we agree with the reviewer that feedforward processing is done primarily within the first 150 ms, and we have updated the statement.

-page 5: “This was confirmed by the observation that more shallow networks benefit more..” . two instances of ‘more’ ; remove the first one.
Page 16: ”For more complex scenes, on the other hand, the first feed-forward sweep might not be not sufficiently informative, …” . The second ‘not’ is unnecessary.
Page 19: “Participants performed on an object recognition task (Figure 1C).” ‘On’ is not needed.

Response: We thank the reviewer for pointing out these typo's that have been corrected.

Cited literature:

Jeurissen, D., Self, M. W., & Roelfsema, P. R. (2016). Serial grouping of 2D-image regions with object-based attention in humans. Elife, 5, e14320.

Kar, K., Kubilius, J., Schmidt, K., Issa, E. B., & DiCarlo, J. J. (2019). Evidence that recurrent circuits are critical to the ventral stream’s execution of core object recognition behavior. Nature neuroscience, 22(6), 974-983.

Kubilius, J., Schrimpf, M., Nayebi, A., Bear, D., Yamins, D. L., & DiCarlo, J. J. (2018). Cornet: Modeling the neural mechanisms of core object recognition. BioRxiv, 408385.

Kubilius, J., Schrimpf, M., Kar, K., Rajalingham, R., Hong, H., Majaj, N., ... & Nayebi, A. (2019). Brain-like object recognition with high-performing shallow recurrent anns. In Advances in Neural Information Processing Systems (pp. 12785-12796).

Liao, Q., & Poggio, T. (2016). Bridging the gaps between residual learning, recurrent neural networks and visual cortex. arXiv preprint arXiv:1604.03640.
